# Intrinsic impacts of the expression of PD-L1 on postoperative recurrence in *EGFR*-mutated lung adenocarcinoma

**DOI:** 10.3389/fonc.2024.1415729

**Published:** 2024-08-30

**Authors:** Atsushi Ito, Shu Kano, Tomohito Tarukawa, Yuta Suzuki, Tadashi Sakaguchi, Kentaro Ito, Yoichi Nishii, Osamu Taguchi, Hiroki Yasui, Motoshi Takao, Osamu Hataji

**Affiliations:** ^1^ Department of Thoracic Surgery, Respiratory Center, Matsusaka Municipal Hospital, Matsusaka, Mie, Japan; ^2^ Department of Thoracic and Cardiovascular Surgery, Mie University Graduate School of Medicine, Tsu, Mie, Japan; ^3^ Department of Respiratory Medicine, Respiratory Center, Matsusaka Municipal Hospital, Matsusaka, Mie, Japan

**Keywords:** lung adenocarcinoma, programmed cell death ligand-1, epidermal growth factor receptor, postoperative recurrence, overall survival

## Abstract

**Objectives:**

This study aimed to assess the intrinsic impacts of the expression of PD-L1 on postoperative recurrence and the prognosis in patients with *epidermal growth factor receptor* (*EGFR*)-mutated lung adenocarcinomas.

**Patients and methods:**

Data from 221 surgically resected pathological stage IA–IIIA lung adenocarcinomas, collected between 2017 and 2019, were analyzed. This included measurements of *EGFR* mutations and the PD-L1 expression. Recurrence-free survival (RFS) and overall survival (OS) were estimated using a Kaplan-Meier analysis and log-rank test. The independent risk factors for RFS were assessed using univariate and multivariate analyses.

**Results:**

Among the patients, 140 were PD-L1-negative (<1%), while 81 were PD-L1-positive (≥1%). PD-L1 positivity was significantly associated with male sex (p=0.038), smoking habit (p=0.005), ND2 lymph node dissection (p=0.013), higher malignant subtype (p=0.003), higher histological grade (p=0.001), and advanced pathological stage (p=0.004). Conversely, *EGFR* mutations were more common in the PD-L1-negative group than in the PD-L1-positive group (p=0.006). Patients were categorized into four groups based on their *EGFR* mutation status and PD-L1 expression status: PD-L1-positive (≥1%) with or without *EGFR* mutations (*EGFR*(+)/PD-L1≥1% or *EGFR (*–)/PD-L1≥1%), and PD-L1-negative (<1%) with or without *EGFR* mutations (*EGFR*(+)/PD-L1<1% or *EGFR (*–)/PD-L1<1%). Among these groups, *EGFR*(+)/PD-L1≥1% cases exhibited the worst 5-year RFS (log-rank, p=0.010), while there was no significant difference in 5-year OS (log-rank, p=0.122). Furthermore, a multivariate analysis revealed that PD-L1 positivity was an independent significant factor for RFS in *EGFR*-mutated lung adenocarcinoma (p=0.013).

**Conclusion:**

PD-L1 positivity emerged as an independent risk factor for RFS in patients with *EGFR*-mutant resected lung adenocarcinoma. These findings may provide valuable insights into the prognostic impact of PD-L1 expression and guide the implementation of postoperative adjuvant therapy in this patient population.

## 1 Introduction

The emergence of molecular-targeted agents and immune checkpoint inhibitors (ICIs) has resulted in a substantial paradigm shift in the perioperative treatment of early-stage non-small cell lung cancer (NSCLC). The phase III ADAURA trial conclusively demonstrated that the administration of adjuvant osimertinib, a third-generation *epidermal growth factor receptor* (*EGFR*) tyrosine kinase inhibitor (TKI), was associated with enhanced postoperative outcomes in patients with pathological stage IB–IIIA *EGFR*-mutated lung adenocarcinoma (1, 2). Furthermore, the phase III IMpower010 trial reported the significant advantages of adjuvant atezolizumab, a programmed death-ligand 1 (PD-L1) antibody, in patients in whom ≥1% of tumor cells were positive for PD-L1 (3, 4). After these pivotal trials, osimertinib and atezolizumab were approved as adjuvant therapies in Japan, with both treatments showing notable benefits in terms of disease-free survival (DFS) and overall survival (OS). Therefore, information on *EGFR* mutations and the expression of PD-L1 is extremely important for the development of postoperative therapeutic strategies.

However, the intrinsic impact of the PD-L1 expression status on postoperative recurrence in *EGFR*-mutated lung adenocarcinoma remains unknown. The expression of PD-L1 has been reported to be upregulated by various factors including oncogenic signaling pathways, tumor mutation burden, and inflammatory cytokines such as interferon-γ (5, 6). In this context, downstream signaling pathways of *EGFR* are known to contribute to the upregulation and activation of PD-L1 (7, 8). Therefore, the PD-L1 expression status may serve as an indicator of the strength of its downstream signaling in *EGFR*-mutated lung adenocarcinoma, resulting in a higher malignant potential.

In this context, the simultaneous analysis of both *EGFR* mutations and PD-L1 expression status in patients with surgically resected lung cancer may lead to the uncovering of recurrence risk cohorts and the guidance of earlier and more intensive adjuvant therapy. Several studies have explored the association between PD-L1 expression and surgical outcomes in patients with *EGFR*-mutated NSCLC. Regarding the expression of PD-L1, Kojima et al. reported that a 1% tumor proportion score (TPS) increases the risk of postoperative recurrence 1.016-fold (9). Additionally, Saw et al. found that PD-L1 positivity independently predicted worse OS and DFS in patients with early stage *EGFR*-mutated NSCLC (10). However, these studies did not provide stage-specific outcomes, particularly for earlier stages such as stages I A and I B.

Therefore, in this study, we explored the intrinsic impacts of the PD-L1 expression status on postoperative recurrence in patients with surgically resected *EGFR*-mutated lung adenocarcinoma, stratified by each stage of the disease, utilizing follow-up data predating adjuvant osimertinib and atezolizumab. To provide background information, we included a comparison cohort consisting of patients with surgically resected wild-type *EGFR* lung adenocarcinoma.

## 2 Methods

### 2.1 Study design

This retrospective, single-center, observational study was conducted at the Respiratory Center of Matsusaka Municipal Hospital in Japan. This study was approved by the Institutional Review Board of Matsusaka Municipal Hospital (approval no. J227-230203-5-3, February 2023).

### 2.2 Study population

Between 1^st^ January 2017 and 31^st^ December 2019, 237 consecutive patients with lung adenocarcinoma underwent surgery with curative intent at the Respiratory Center of Matsusaka Municipal Hospital. Among them, we enrolled 221 patients who were diagnosed with pathological stage IA-IIIA lung adenocarcinoma and in whom both the *EGFR* mutation and PD-L1 expression status were evaluated. These assessments were typically conducted preoperatively or immediately postoperatively. To avoid potential bias related to recurrence status, cases assessed with the *EGFR* mutation and PD-L1 expression status post-recurrence were excluded from this study. In the case of 11 patients with synchronous multiple lung cancers, one of the highest-grade tumors was included in the analysis. The histopathological diagnosis according to the 8^th^ TNM classification (11) was determined by pathologists. Adjuvant chemotherapy was administered to eligible patients who provided their informed consent according to the guidelines of the Japanese Lung Cancer Association. Clinicopathological features, including age, sex, smoking status, surgical procedure, pathological findings, and the presence of postoperative recurrence and death, were collected from medical records.

### 2.3 Sample processing

Samples were processed using methods previously described by our group (7, 12). Briefly, small tumor tissue samples for the preoperative diagnosis obtained by a computed tomography (CT)-guided percutaneous needle biopsy and endobronchial biopsy were promptly immersed in 10% neutral buffered formalin (NBF) and fixed at room temperature for 12-18 hours. Pulmonary resection samples without a preoperative diagnosis were stored in a refrigerator at 4°C for less than 3 h after sampling for an intraoperative rapid diagnosis (IRD) and then placed in 10% NBF for 24-48 h at room temperature. Formalin-fixed tissues were embedded in paraffin to prepare formalin-fixed paraffin-embedded (FFPE) blocks. To measure *EGFR* mutations and PD-L1 status, five slides of 5μm thick tissue sections from small biopsy and surgical resection samples were submitted to the central laboratory of LSI Medicine Laboratories (Tokyo, Japan).

### 2.4 *EGFR* mutation assessment

All *EGFR* tests were performed using the tumor tissue. DNA was extracted from FFPE samples using a QIAamp DNA FFPE Tissue kit (Qiagen, Hilden, Germany). *EGFR* mutations were prospectively detected using the PNA-LNA PCR clamp test at the central laboratory of the LSI Medicine Laboratories. Exon 18 (p.G709X and p.G719A/C/S), exon 19 (deletion and insertion), exon 20 (p.S768I, p.T790M and insertion), and exon 21 (p.L833X, p.L858R, and p.L861Q), and other mutations in exons 18-21 (p.V769M, p.V834L, p.K860I, etc) have been identified (12). p.S768I, exon 20 insertion, and p.L833X have been included in the PNA-LNA PCR clamp test reports since October 2019 (12). We classified exon 19 deletion (Ex19del) and exon 21 p.L858R (L858R) as common mutations, whereas other *EGFR* mutations and compound mutations were classified as uncommon mutations.

### 2.5 PD-L1 immunohistochemical staining and scoring

Immunohistochemical staining for PD-L1 was performed using the 22C3 pharmDx assay (Santa Clara, CA, USA) in formalin-fixed tumor samples obtained by surgical resection or small biopsy. The PD-L1 expression was determined using the TPS, which is defined as the percentage of viable tumor cells showing partial or complete membrane staining. Based on PD-L1 expression, the tumors were categorized into two groups, negative (PD-L1 < 1%) and positive (PD-L1≥1%), according to the TPS, by counting at least 100 viable cells.

### 2.6 Statistical analysis

The baseline characteristics of the different groups were compared using the chi-square test. The clinical outcomes were assessed using RFS and OS. RFS was defined as the time from surgery to tumor recurrence or the date of the last follow-up examination. OS was defined as the time from the date of surgery to death from any cause. Survival curves were estimated using the Kaplan-Meier method. Differences in survival curves were assessed using the log-rank test. Univariate and multivariate Cox regression analyses were performed to assess the association between clinicopathological characteristics and RFS. All analyses were performed using SPSS (version 29.0; SPSS Inc., Chicago, IL, USA), and values of p<0.05 were considered to indicate statistical significance.

## 3 Results

### 3.1 Patient characteristics

**Figure 1:**
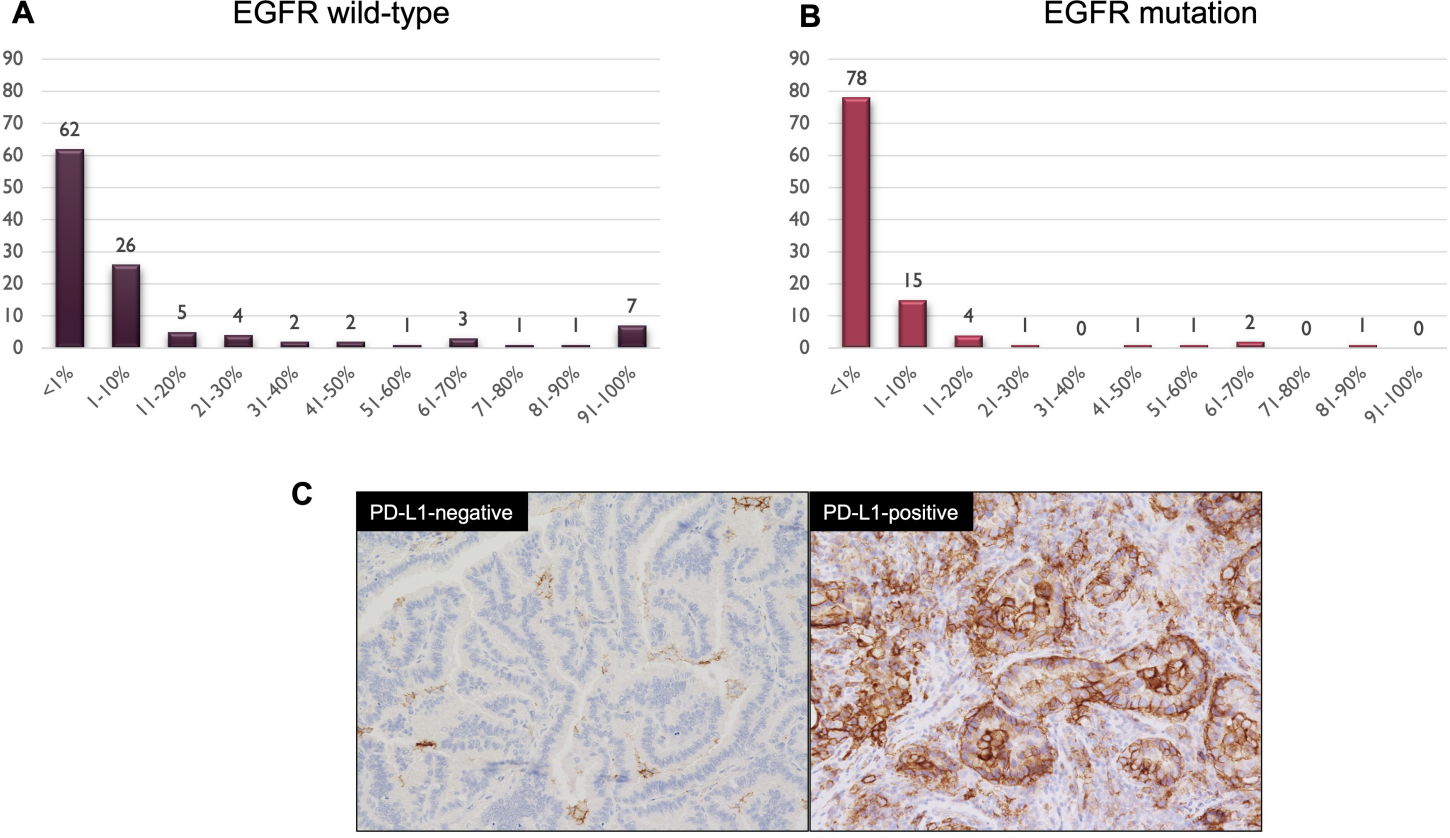
The programmed death-ligand 1 (PD-L1) expression profiles of patients with *epidermal growth factor receptor* (*EGFR*) wild-type **(A)** and *EGFR* mutation **(B)**. Four patients with PD-L1 expression assessed in approximately 1-49% were excluded from these profiles. The representative images of PD-L1 immunohistochemical staining using the 22C3 pharmDx assays (left: PD-L1-negative [0%], right: PD-L1-positive [95%], magnification: ×200) **(C)**.

A total of 237 consecutive patients with stage IA-IIIA lung adenocarcinoma underwent lung resection at our hospital. As 16 patients, whose *EGFR* and PD-L1 statuses were not examined, were excluded, we reviewed 221 patients in our study. The PD-L1 expression profiles of these patients were shown in **Figures 1A, B**. Compared to patients with *EGFR* mutation, those with *EGFR* wild-type exhibited a relatively higher PD-L1 expression status. To clarify our criteria of PD-L1 expression status, we presented representative immunohistochemical images showing PD-L1-negative and PD-L1-positive in **Figure 1C**. As there were only 4 patients with PD-L1 ≥ 50% among the *EGFR* mutation cases, we classified the 221 patients into two distinct groups: the PD-L1-negative (<1%) group (n=140) and the PD-L1-positive (≥1%) group (n=81). The patient characteristics are summarized in **Table 1**.

**Table 1:** Clinicopathological characteristics of patienst with surgically resected lung adenocarcinoma.

Characteristics	Total	Negative-PD-L1 (<1%)	Positive-PD-L1 (≥1%)	*p* value
	(n=221)	(n=140)	(n=81)	
Age, median (range)	73 (30-92)	73 (30-92)	73 (39-89)	0.718
Sex, n (%)				**0.038**
Male	108 (48.9)	61 (43.6)	47 (58.0)	
Female	113 (51.1)	79 (56.4)	34 (42.0)	
Smoking Status, n (%)				**0.005**
Never	104 (47.1)	76 (54.3)	28 (34.6)	
Former+Current	117 (52.9)	64 (45.7)	53 (65.4)	
Surgical Procedure, n (%)				0.075
Partial resection	56 (25.3)	43 (30.7)	13 (16.0)	
Segmentectomy	18 (8.1)	9 (6.4)	9 (11.1)	
Lobectomy (+ partial resection)	143 (64.7)	85 (60.7)	58 (71.6)	
Bilobectomy	4 (1.8)	3 (2.1)	1 (1.2)	
Lymph node dissection, n (%)				**0.013**
ND0	60 (27.1)	47 (33.6)	13 (16.0)	
ND1	22 (9.9)	11 (7.6)	11 (13.6)	
ND2	139 (62.9)	82 (58.6)	57 (70.4)	
Histologic subtype, n (%)				**0.003**
Lepidic	66 (22.9)	49 (35.0)	17 (20.9)	
Papillary	112 (50.7)	74 (52.9)	38 (46.9)	
Acinar	8 (3.6)	4 (2.9)	4 (4.9)	
Solid	22 (9.9)	7 (5.0)	15 (18.5)	
Micropapillary	6 (2.7)	2 (1.4)	4 (4.9)	
Unknown	7 (3.2)	4 (2.9)	3 (3.7)	
Pleural invasion, n (%)				0.362
No	143 (64.7)	89 (63.6)	54 (66.7)	
Yes	31 (14.0)	15 (10.7)	16 (19.8)	
Unknown	47 (21.3)	36 (25.7)	11 (13.6)	
Grade, n (%)				**0.001**
Well	124 (56.1)	91 (65.0)	33 (40.7)	
Moderate	79 (35.7)	40 (28.6)	39 (48.1)	
Poor	4 (1.8)	1 (0.7)	3 (3.7)	
Unknown	14 (6.3)	8 (5.7)	6 (7.4)	
Pathological Stage, n (%)				**0.004**
IA1	81 (36.6)	62 (44.3)	19 (23.5)	
IA2	55 (24.9)	27 (19.3)	28 (34.6)	
IA3	21 (9.5)	14 (10.0)	7 (8.6)	
IB	30 (13.6)	15 (10.7)	15 (18.5)	
IIA	4 (1.8)	4 (2.9)	0 (0.0)	
IIB	16 (7.2)	12 (8.6)	4 (4.9)	
III	14 (6.3)	6 (4.3)	8 (9.8)	
Adjuvant Therapy, n (%)				0.285
None	182 (82.4)	119 (85.0)	63 (77.8)	
Oral anticancer drug (tegafur/uracil)	19 (8.6)	9 (6.4)	10 (12.3)	
Platinum-based chemotherapy	20 (9.0)	12 (8.6)	8 (9.8)	
EGFR status, n (%)				**0.006**
Common mutation (Ex19del and L858R))	96 (43.4)	71 (50.7)	25 (30.9)	
Uncommon mutation	9 (4.1)	7 (5.0)	2 (2.4)	
Wild-type	116 (52.5)	62 (44.3)	54 (66.7)	

n, number; PD-L1, programmed death-ligand 1; EGFR, epidermal growth factor receptor; Ex19del, exon 19 deletion; L858R, exon 21 p.L858R. Bold values indicate statistically significant.

The median age of the patients was 73 years (30–92). Common *EGFR* mutations were detected in 96 of 221 patients with lung adenocarcinoma (43.4%), and uncommon *EGFR* mutations were detected in 9 of 221 patients (4.1%). The PD-L1-negative group and the PD-L1-positive group were not significantly different in terms of age (p=0.718), surgical procedure (p=0.075), pleural invasion (p=0.362), and the percentage of adjuvant therapy (p=0.285). However, PD-L1 positivity was significantly associated with male sex (p=0.038), smoking habit (p=0.005), ND2 lymph node dissection (p=0.013), higher malignant subtype (p=0.003), higher histological grade (p=0.001), advanced pathologic stage (p=0.004). In contrast, *EGFR* mutations were more frequent in the PD-L1-negative group than in the PD-L1-positive group (p=0.006).

### 3.2 Prognostic impacts of the PD-L1 expression status

**Figure 2:**
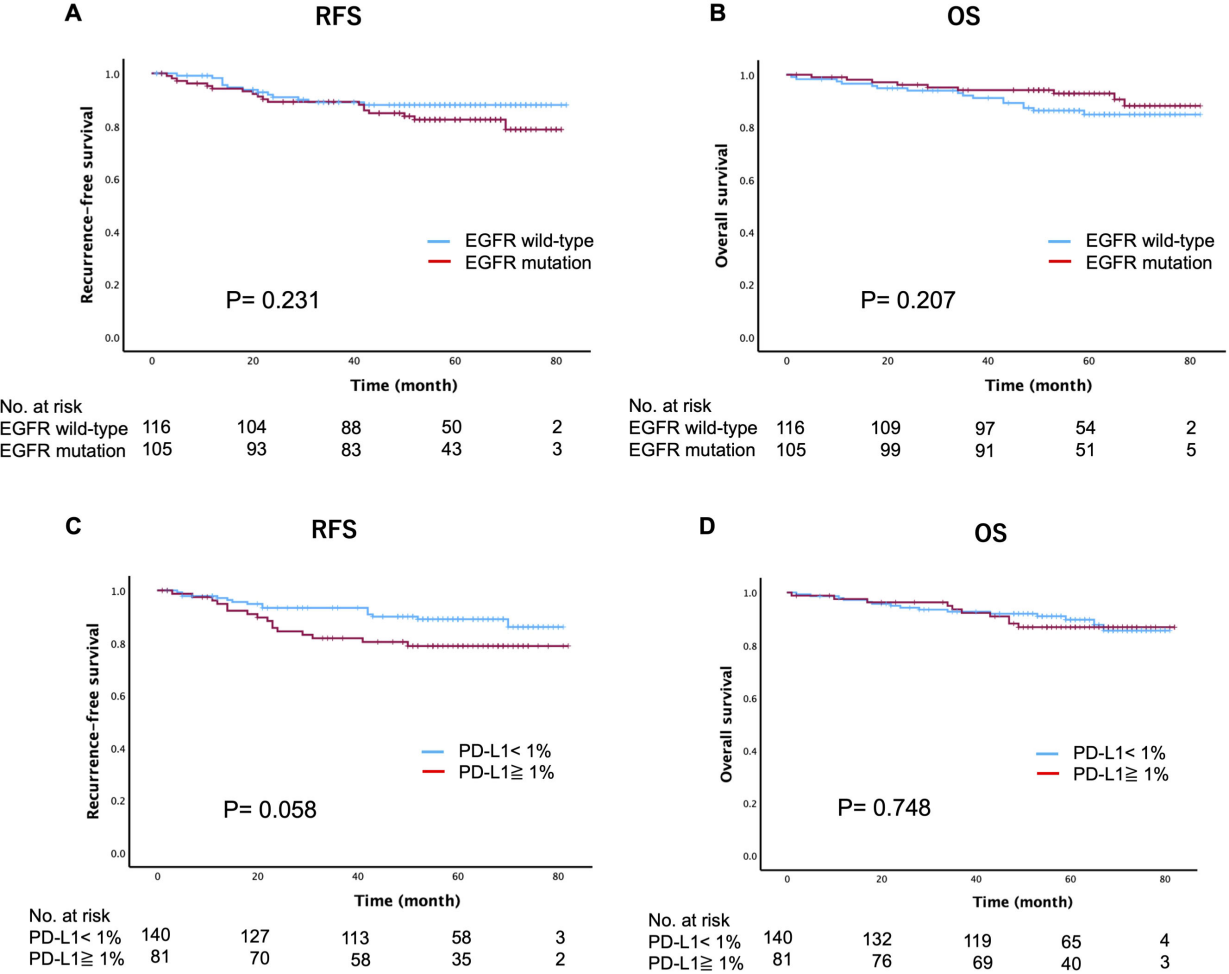
Kaplan-Meier estimates of recurrence-free survival and overall survival based on the *epidermal growth factor receptor* (*EGFR*) mutation status **(A, B)** and programmed death-ligand 1 (PD-L1) expression status **(C, D)**.

At a median follow-up of 52 months (range 1-82), recurrence was observed in 31 patients (14%) and death occurred in 25 patients (11.3%). The Kaplan-Meier curves for RFS and OS based on the *EGFR* mutation or PD-L1 expression status are shown in **Figure 2**. Intriguingly, *EGFR* mutations were not significantly associated with recurrence (p=0.231) or death (p=0.207). However, patients with *EGFR* mutations exhibited worse 5-year RFS in comparison to those with wild-type *EGFR*, while the 5-year OS was better in patients with *EGFR* mutations (*EGFR* mutation vs. *EGFR* wild-type: RFS, 82.4% vs. 88%; OS, 92.8% vs. 84.8%). When *EGFR* mutations were converted into specific mutation subtypes (Ex19del, L858R, and uncommon mutations), no significant differences were observed in terms of recurrence (p=0.178) or death (p=0.528) (**Supplementary Figure 1**). Regarding the assessment of the PD-L1 expression status, PD-L1-positive (≥1%) cases showed a trend towards worse 5-year RFS in comparison to PD-L1-negative (<1%) cases, although this difference was not statistically significant (p=0.058). Additionally, the PD-L1 expression status had no significant impact on 5-year OS (p=0.748) (PD-L1-positive vs. PD-L1-negative: RFS, 89% vs. 78.8%; OS, 92.8% vs. 84.8%).

**Figure 3:**
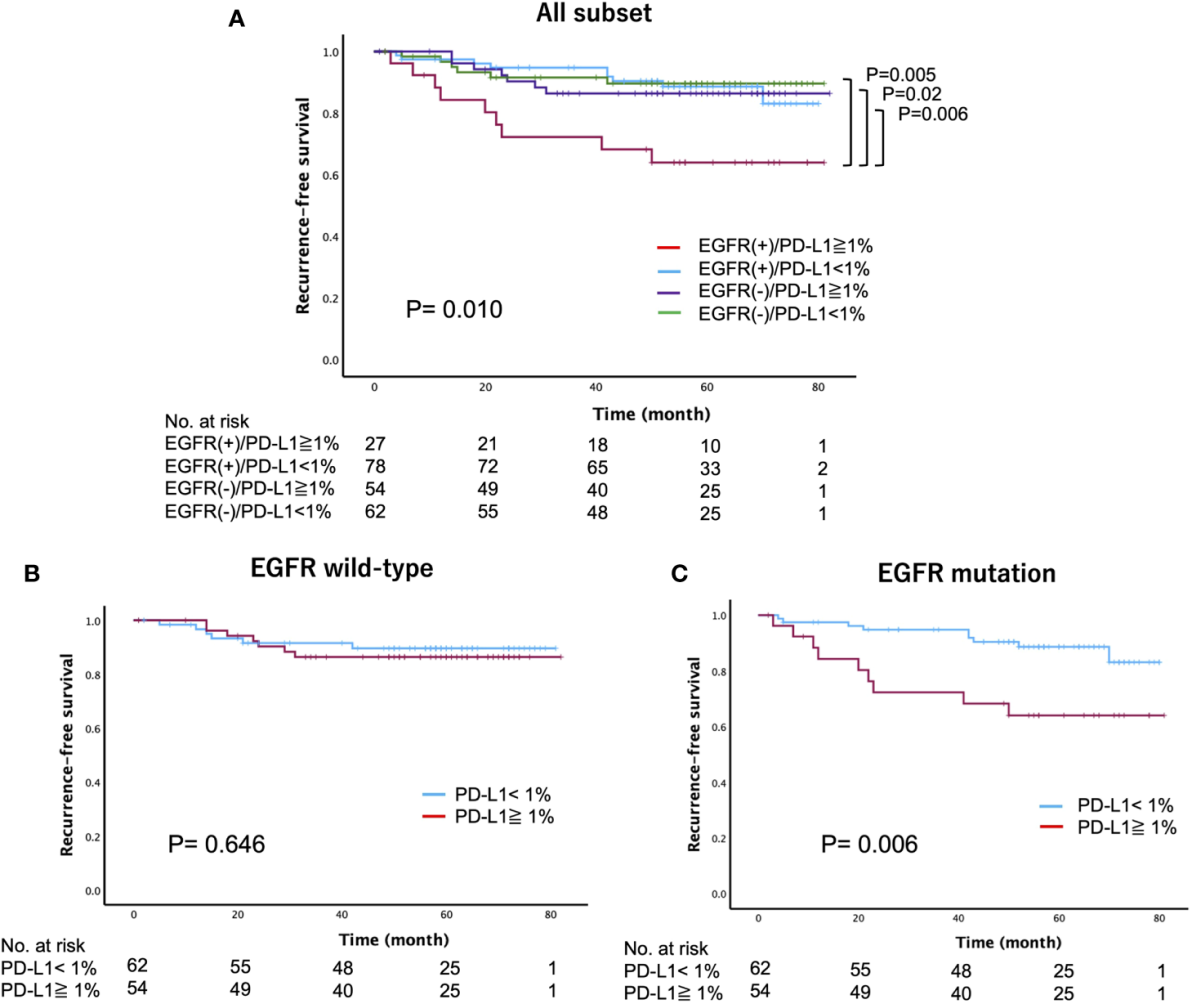
Recurrence-free survival stratified by the *epidermal growth factor receptor* (*EGFR*) mutation and programmed death-ligand 1 (PD-L1) expression status in patients with surgically resected lung adenocarcinoma **(A)** all subset, **(B)** *EGFR* wild-type, **(C)** *EGFR* mutation).

**Figure 4:**
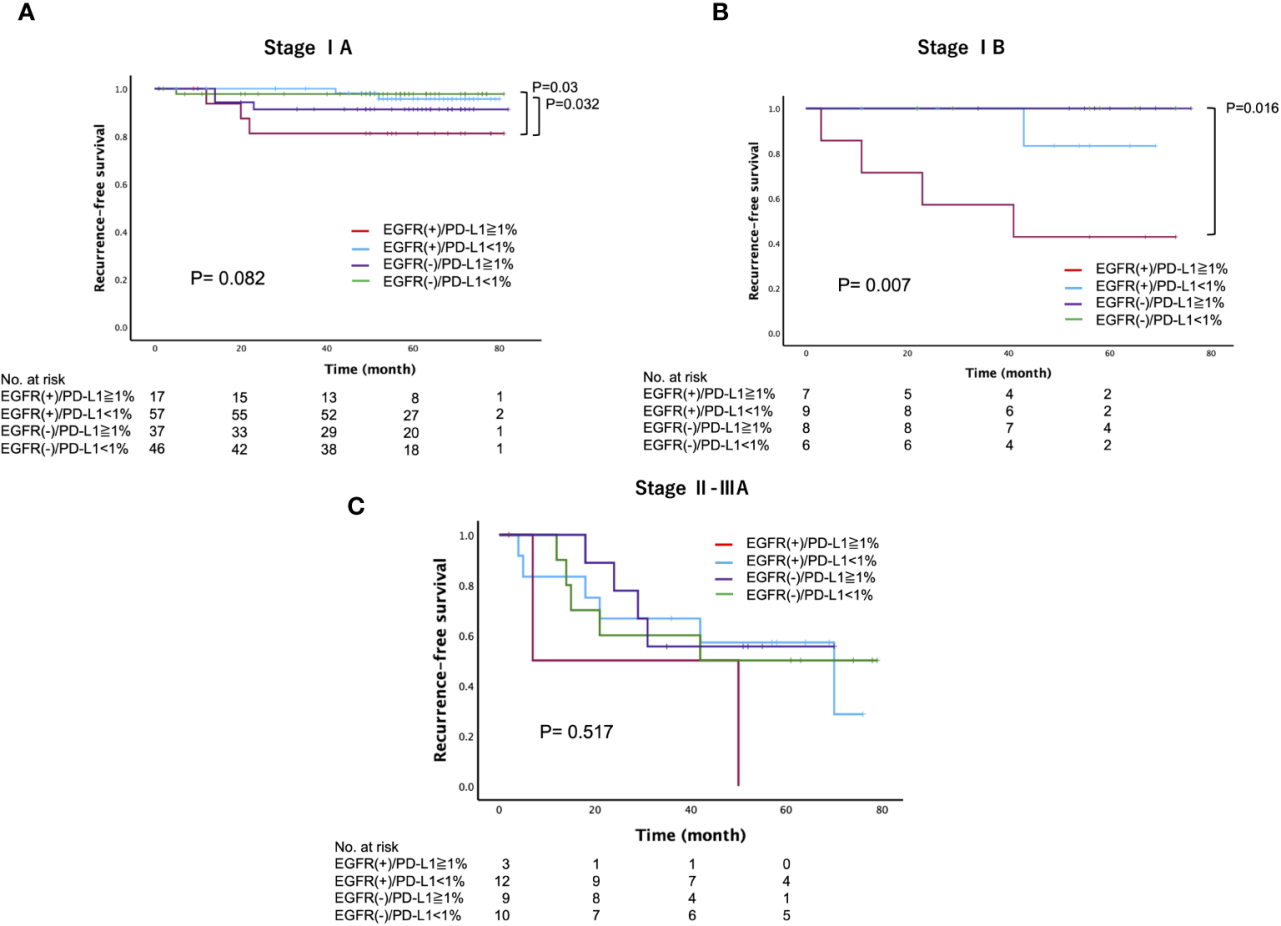
Recurrence-free survival, stratified by the *epidermal growth factor receptor* (*EGFR*) mutation and programmed death-ligand 1 (PD-L1) expression status across different disease stages **(A)** Stage IA, **(B)** Stage IB, **(C)** Stage II-IIIA) in patients with surgically resected lung adenocarcinoma.

We further investigated whether the PD-L1 expression status has intrinsic implications for postoperative recurrence in patients with surgically resected *EGFR*-mutated lung adenocarcinoma. Specifically, we categorized patients into four groups based on their *EGFR* mutation status and PD-L1 expression status: PD-L1-positive (≥1%) cases with or without *EGFR* mutations (*EGFR*(+)/PD-L1≥1% or *EGFR (*–)/PD-L1≥1%), and PD-L1-negative (<1%) cases with or without *EGFR* mutations (*EGFR*(+)/PD-L1<1% or *EGFR (*–)/PD-L1<1%). Among these groups, we found that *EGFR*(+)/PD-L1≥1% of cases had the worst 5-year RFS (64%), followed by *EGFR (*–)/PD-L1≥1% (86.4%), *EGFR*(+)/PD-L1<1% (88.6%), and *EGFR (*–)/PD-L1<1% (89.6%) (log-rank, p=0.010) (**Figure 3A**). However, there was no significant difference in the 5-year OS (log-rank, p=0.122) (**Supplementary Figure 2**). When RFS was compared according to *EGFR* status, we found a significant difference in RFS between PD-L1-positive and PD-L1-negative cases in patients with *EGFR*-mutated lung adenocarcinoma (log-rank, p=0.006), but not in patients with *EGFR* wild-type lung adenocarcinoma (log-rank, p=0.646) (**Figures 3B, C**). Subsequently, we compared the 5-year RFS of these four categories stratified by disease stage. The results revealed a similar trend across all disease stages, with the worst 5-year RFS observed in the *EGFR*(+)/PD-L1≥1% cases, particularly in stage IB (log-rank, stage IA: p=0.082, stage IB: p=0.007, stage II-IIIA: p=0.517) (**Figure 4**).

### 3.3 Univariate and multivariate analyses

We evaluated factors related to postoperative tumor recurrence among cases with *EGFR* mutations. The results of the univariate and multivariate Cox proportional hazards analyses of factors associated with RFS are presented in **Table 2**. In the univariate analysis, histologic subtype (HR, 11.2; 95% CI, 1.4-84.5), tumor grade (HR, 5.1; 95% CI, 1.6-16.4), pathologic stage (HR, 11.6; 95% CI, 3.7-36.1), and PD-L1 status (HR, 3.1; 95% CI, 1.1-8.0) were significantly associated with RFS. A multivariate analysis incorporating these four factors that showed statistical significance in the univariate analysis revealed that the pathologic stage (HR, 5.4; 95% CI, 1.5-18.2) and PD-L1 status (HR, 3.9; 95% CI, 1.3-11.6) were significantly associated with postoperative recurrence.

**Table 2:** Univariate and Multivariate analyses for recurrence free survival among the patients with EGFR-mutated lung adenocarcinoma.

Varibles	Univariate HR (95%CI)	P value	Multivariate HR (95%CI)	P value
Age (65< vs ≥65)	1.992 (0.455-8.719)	0.36	NA	–
Sex (Male vs Female)	0.755 (0.278-2.048)	0.581	NA	–
Smoking history (No vs Yes)	0.728 (0.236-2.246)	0.58	NA	–
Subtype (Lepidic vs Papillary/Acinar/Solid/Micropapillary)	11.213 (1.487-84.547)	**0.019**	2.546 (0.514-12.606)	0.252
Tumor Grade (Well vs Moderate/Poorly)	5.145 (1.610-16.440)	**0.006**	1.046 (0.286-3.828)	0.946
Pathological stage (IA vs IB-IIIA)	11.627 (3.736-36.182)	**<0.001**	5.404 (1.598-18.270)	**0.007**
PD-L1 status (Negative[<1%] vs Positive[≥1%])	3.102 (1.195-8.054)	**0.037**	3.953 (1.339-11.671)	**0.013**

EGFR, epidermal growth factor receptor; PD-L1, programmed death-ligand 1; HR, hazard ratio; CI, confidential index; vs, versus; NA, not applicable. Bold values indicate statistically significant.

## 4 Discussion

We routinely conducted *EGFR* mutation assessments and evaluated the PD-L1 status in all patients with surgically resected lung adenocarcinoma. This study focused on analyzing postoperative recurrence and the prognosis in 221 consecutive patients with lung adenocarcinoma, considering both *EGFR* mutations and the PD-L1 expression status. To the best of our knowledge, this is the first report to investigate the intrinsic impacts of the PD-L1 expression status on postoperative recurrence among *EGFR*-mutated lung adenocarcinomas with a specific emphasis on stage-related outcomes. In our Kaplan-Meier analysis, *EGFR*(+)/PD-L1≥1% was significantly associated with worse RFS regardless of pathological stage. Furthermore, both univariate and multivariate analyses identified PD-L1 expression ≥1% as a significant prognostic factor for RFS in patients with *EGFR*-mutated lung adenocarcinoma. This study will contribute to identifying cohorts with an increased risk of postoperative recurrence, based on both the *EGFR* mutation and PD-L1 expression status, among patients with surgically resected lung adenocarcinoma.

*EGFR* is reported to be the most frequently altered driver gene of lung adenocarcinoma in Asian patients, with an incidence ranging from 40% to 50% (13). Two common types of *EGFR* mutations, L858R and Ex19 del, are prevalent oncogenic driver mutations (13). These common *EGFR* mutations have become a clinically important molecular subset because patients with tumors harboring common *EGFR* mutations benefit from *EGFR*-TKI treatment not only in the management of metastatic or advanced disease but also in the postoperative adjuvant setting. The phase III ADAURA trial demonstrated that, in comparison to placebo, adjuvant osimertinib led to significantly longer overall survival among patients with completely resected, *EGFR*-mutated NSCLC (HR 0.49; 95%CI, 0.34-0.70) (2). Among these patient cohorts, postoperative recurrence was less frequent in patients who received osimertinib (27%) than in those who received a placebo (60%) (2).

However, despite the remarkable prognostic benefits offered by osimertinib, a population of patients with *EGFR*-mutated lung adenocarcinoma who are at high risk for postoperative recurrence has not yet been clearly identified. Yang et al. examined the association between *EGFR* mutations and postoperative recurrence based on radiological appearance, including ground-glass nodules (GGNs) and pure-solid nodules in clinical-stage lung adenocarcinoma (14). Interestingly, *EGFR* mutations were associated with worse RFS and a higher incidence of distant recurrence in patients with a radiologic pure-solid appearance, but not in patients with GGNs. Similarly, Ito et al. suggested that the prognostic impact of *EGFR* mutations should be considered together with the pathological stage and histological subtype (15). Typically, *EGFR* mutations are more frequently detected in patients with early-stage disease with predominant lepidic lesions, such as adenocarcinoma *in situ* (AIS). Since these lesions are associated with little or no risk of recurrence (16), it may be appropriate to exclude these earlier-stage diseases from recurrence and prognostic analyses. Therefore, in our study, we focused on pathological stage IA–IIIA lung adenocarcinoma and excluded cases with AIS.

The expression of PD-L1 is upregulated by the signaling pathways downstream of *EGFR* mutations, such as the mitogen-activated protein kinases (MAPK)/extracellular signal-regulated kinase (ERK), Janus kinase (JACK)/signal transducer and activator of transcription 3 (STAT3) signaling pathways (7, 8). Studies of *EGFR*-mutated cell lines have shown higher levels of PD-L1 in comparison to wild-type *EGFR* cells, with *EGFR* pathway activation leading to an increase in the expression of PD-L1 (17). Therefore, the expression of PD-L1 may indicate high malignant potential through the strength of its downstream signaling in *EGFR*-mutated lung adenocarcinoma. In addition, Chen et al. reported that PD-L1 activation mediated by *EGFR* induced the apoptosis of T cells through PD-L1/PD-1 axis *in vitro* co-culture systems (18). This finding suggests that PD-L1 expression of *EGFR*-mutated lung adenocarcinoma might impair the function of T cells within the tumor microenvironment, potentially leading to cancer recurrence. Indeed, our results showed that PD-L1 positive cases have worse RFS than PD-L1 negative cases in *EGFR*-mutated lung adenocarcinoma, but not in *EGFR*-wildtype lung adenocarcinoma (**Figures 3B, C**).

Despite experiencing a higher recurrence rate, patients with *EGFR*-mutated lung adenocarcinoma exhibited relatively better overall survival. This favorable outcome could be attributed to subsequent administration of *EGFR*-TKIs following postoperative recurrence, which was observed in 68.4% (13/19) of patients with *EGFR* mutations, resulting in a prolonged prognosis (**Supplementary Figure 3**). However, the underlying reason for the favorable prognosis in PD-L1-positive cases in comparison to PD-L1-negative cases in *EGFR*-mutated lung adenocarcinoma remains unclear. In contrast to our results, Saw et al. demonstrated inferior outcomes in patients with PD-L1-positive *EGFR*-mutated lung adenocarcinoma (10). Similarly, the Osi-fact trial reported poorer response to *EGFR*-TKI in PD-L1-positive cases compared to PD-L1-negative cases in *EGFR*-mutated NSCLC (19). However, it is important to note that this trial included only about 30% of recurrence cases, with Stage IV metastatic lung cancer accounting for 65.8%. Given that postoperative recurrence generally has a better prognosis than Stage IV metastatic lung cancer, the results of the Osi-Fact trial should not be directly applied to cases of postoperative recurrence. Currently, there is no consensus on whether *EGFR*-TKIs or ICIs should be used as adjuvant therapy for *EGFR*(+)/PD-L1≥1% groups, particularly in cases with the overexpression of PD-L1 (≥50%). ICIs have been reported to have limited efficacy in the metastatic setting for *EGFR*-mutated NSCLC, regardless of PD-L1 score (20). In our study, only one patient with recurrent *EGFR*-mutated lung adenocarcinoma received ICIs before *EGFR*-TKI therapy (**Supplementary Figure 3**). Therefore, we cannot conclude that the preferential administration of ICIs improved the prognosis of the *EGFR*(+)/PD-L1≥1% group. Future research is needed to clarify the discrepancies and to gain a better understanding of the impact of the expression of PD-L1 on the prognosis of this patient population.

This study was associated with several limitations. First, there is a potential selection bias because this was a single-center retrospective study. Second, the relatively small sample size, particularly in the analysis of 5-year RFS stratified by disease stage, may have influenced the statistical power and precision of the results. Although we attempted further stratification within the stage IA cohort into subgroups, such as stage IA1, IA2, and IA3, the limited number of patients posed a challenge. Larger-scale studies, ideally conducted on a nationwide basis, are required to validate our findings. Thirdly, the inclusion of patients who received postoperative adjuvant chemotherapy may have confounded our results. While adjuvant chemotherapy could affect recurrence and the prognosis, the distribution of patients receiving chemotherapy was comparable across the different PD-L1 expression status groups.

## 5 Conclusions

PD-L1 positivity is an independent risk factor for RFS in patients with *EGFR*-mutated lung adenocarcinoma. Our findings may help us understand the impact of PD-L1 expression status on prognosis and how to better implement postoperative adjuvant therapy for these patients.

## Data Availability

The raw data supporting the conclusions of this article will be made available by the authors, without undue reservation.

## Supplementary material

Supplementary Figure 1 Kaplan-Meier estimates of recurrence-free survival **(A)** and overall survival **(B)** stratified by *epidermal growth factor receptor* (*EGFR*)-specific mutation subtypes (Ex19del, L858R, and uncommon mutations) and wild-type *EGFR*.

Supplementary Figure 2 Overall survival stratified by the programmed death-ligand 1 (PD-L1) expression status in patients with wild-type *epidermal growth factor receptor* (*EGFR*).

Supplementary Figure 3 Swimmer plots of survival in *EGFR*-mutated lung adenocarcinoma with postoperative recurrence. Deep-colored bars represent the time from surgery to recurrence, while right-colored bars represent the survival time after recurrence. Treatment after recurrence is depicted at the tail of the bars, with triangles indicating postoperative recurrence, and crosses indicating death. Abbreviations: *EGFR*-TKI, epidermal growth factor receptor tyrosine kinase inhibitor; ICI, immune checkpoint inhibitor; BSC, best supportive care; RT, radiation therapy.
