# Supplementary figures and images for: Intrinsic impacts of the expression of PD-L1 on postoperative recurrence in EGFR-mutated lung adenocarcinoma
Source: Front Oncol. 2024 Aug 30;14:1415729. doi: 10.3389/fonc.2024.1415729 (PMC11392724; doi:10.3389/fonc.2024.1415729)

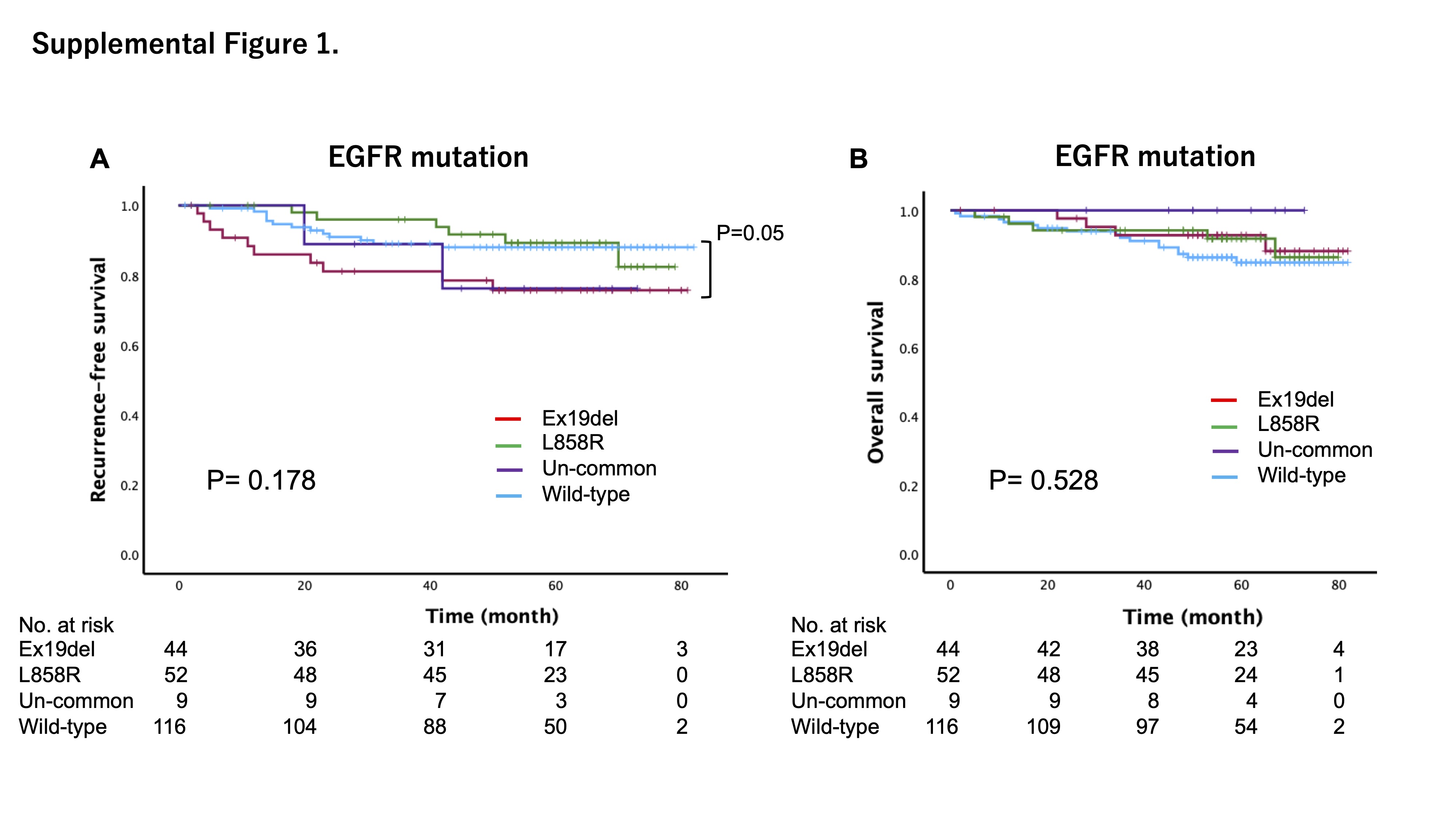

Supplement: Supplementary Figure 1 — Kaplan-Meier estimates of recurrence-free survival (A) and overall survival (B) stratified by epidermal growth factor receptor (EGFR)-specific mutation subtypes (Ex19del, L858R, and uncommon mutations) and wild-type EGFR. [file Image1.jpeg]

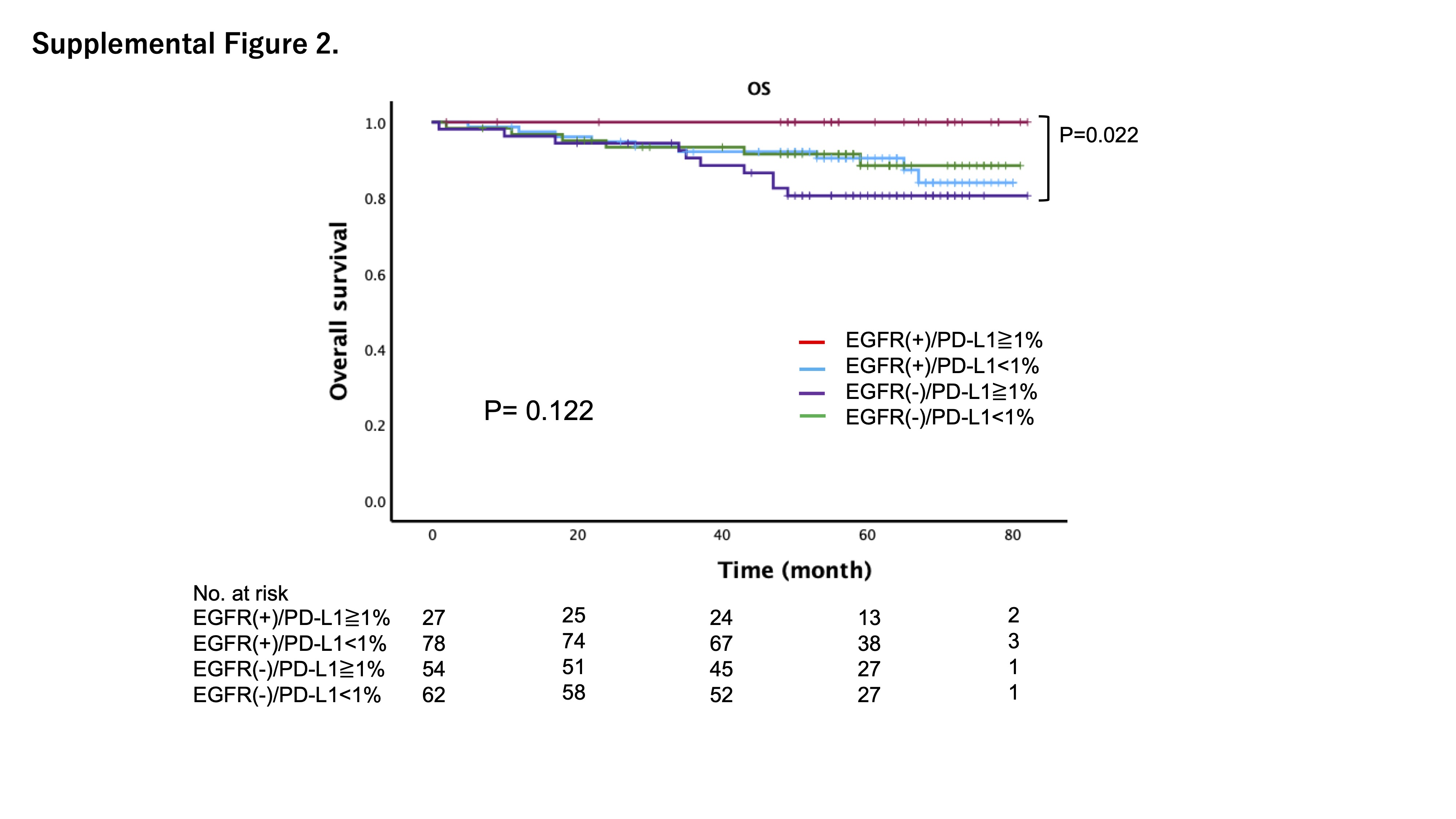

Supplement: Supplementary Figure 2 — Overall survival stratified by the programmed death-ligand 1 (PD-L1) expression status in patients with wild-type epidermal growth factor receptor (EGFR). [file Image2.jpeg]

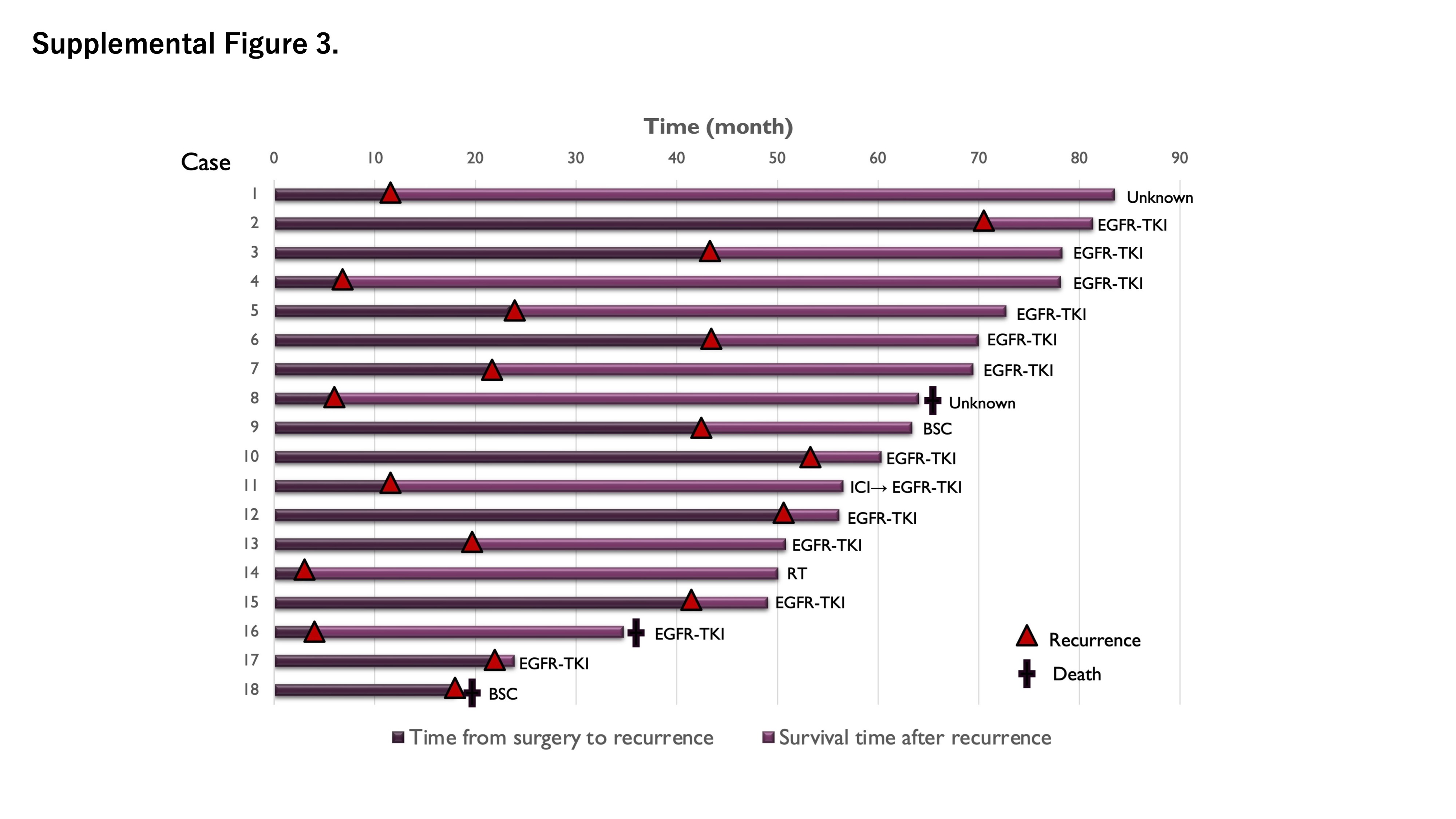

Supplement: Supplementary Figure 3 — Swimmer plots of survival in EGFR-mutated lung adenocarcinoma with postoperative recurrence. Deep-colored bars represent the time from surgery to recurrence, while right-colored bars represent the survival time after recurrence. Treatment after recurrence is depicted at the tail of the bars, with triangles indicating postoperative recurrence, and crosses indicating death. Abbreviations: EGFR-TKI, epidermal growth factor receptor tyrosine kinase inhibitor; ICI, immune checkpoint inhibitor; BSC, best supportive care; RT, radiation therapy. [file Image3.jpeg]
